# Tissues from equine cadaver ligaments up to 72 hours of post-mortem: a promising reservoir of stem cells

**DOI:** 10.1186/s13287-015-0250-7

**Published:** 2015-12-18

**Authors:** Mohamad Khir Shikh Alsook, Annick Gabriel, Joëlle Piret, Olivier Waroux, Céline Tonus, Delphine Connan, Etienne Baise, Nadine Antoine

**Affiliations:** Anatomy Unit, FARAH Research Center & Faculty of Veterinary Medicine, University of Liège, Liège, Belgium; Histology Unit, FARAH Research Center & Faculty of Veterinary Medicine, University of Liège, Liège, Belgium; Embryology Unit, FARAH Research Center & Faculty of Veterinary Medicine, University of Liège, Liège, Belgium; Embryology Unit, GIGA-Development, Stem Cells and Regenerative Medicine and Faculty of Veterinary Medicine, University of Liège, Liège, Belgium; FARAH Research Center & Faculty of Veterinary Medicine, University of Liège, Liège, Belgium

**Keywords:** Equine, Cadaver, Ligament, Mesenchymal stem cells, OCT-4, SSEA-1, TUJ-1, GFAP, TEM

## Abstract

**Background:**

Mesenchymal stem cells (MSCs) harvested from cadaveric tissues represent a promising approach for regenerative medicine. To date, no study has investigated whether viable MSCs could survive in cadaveric tissues from tendon or ligament up to 72 hours of post-mortem. The purpose of the present work was to find out if viable MSCs could survive in cadaveric tissues from adult equine ligaments up to 72 hours of post-mortem, and to assess their ability (i) to remain in an undifferentiated state and (ii) to divide and proliferate in the absence of any specific stimulus.

**Methods:**

MSCs were isolated from equine cadaver (EC) suspensory ligaments within 48–72 hours of post-mortem. They were evaluated for viability, proliferation, capacity for tri-lineage differentiation, expression of cell surface markers (CD90, CD105, CD73, CD45), pluripotent transcription factor (OCT-4), stage-specific embryonic antigen-1 (SSEA-1), neuron-specific class III beta-tubulin (TUJ-1), and glial fibrillary acidic protein (GFAP). As well, they were characterized by transmission electron microscope (TEM).

**Results:**

EC-MSCs were successfully isolated and maintained for 20 passages with high cell viability and proliferation. Phase contrast microscopy revealed that cells with fibroblast-like appearance were predominant in the culture. Differentiation assays proved that EC-MSCs are able to differentiate towards mesodermal lineages (osteogenic, adipogenic, chondrogenic). Flow cytometry analysis demonstrated that EC-MSCs expressed CD90, CD105, and CD73, while being negative for the leukocyte common antigen CD45. Immunofluorescence analysis showed a high percentage of positive cells for OCT-4 and SSEA-1. Surprisingly, in absence of any stimuli, some adherent cells closely resembling neuronal and glial morphology were also observed. Interestingly, our results revealed that approximately 15 % of the cell populations were TUJ-1 positive, whereas GFAP expression was detected in only a few cells. Furthermore, TEM analysis confirmed the stemness of EC-MSCs and identified some cells with a typical neuronal morphology.

**Conclusions:**

Our findings raise the prospect that the tissues harvested from equine ligaments up to 72 hours of post-mortem represent an available reservoir of specific stem cells. EC-MSCs could be a promising alternative source for tissue engineering and stem cell therapy in equine medicine.

## Background

In equine clinical medicine, adult mesenchymal stem cells (MSCs) are commonly used for regeneration of injured tissues [1–3]. MSCs have been found in many tissues wherein quiescent stem cells can become active following injury, migrating into the damaged area to perform repairing functions [4]. MSCs from bone marrow (BM-MSCs) are most generally considered as the universal source in spite of the presence of some detrimental features, such as pain, morbidity and extremely low amounts (0.01–0.001 %) of cells at harvest. Furthermore, a high degree of viral infection and a decreased incidence of proliferation/differentiation with increased aging have been recorded [5–7]. Additionally, it is associated with pneumopericardium in the donor horse secondary to a sternal bone marrow aspiration [8]. As an alternative, adipose-derived mesenchymal stem cells (AD-MSCs) have also been used for regenerative therapies. Although relatively easier to recover, nevertheless the current evidence shows that the AD-MSCs appear less effective than BM-MSCs in differentiation assays and generate a higher risk of donor-site morbidity in comparison with BM-MSCs [9]. These hindrances justify an investigation into other sources that could be used for therapeutic benefit [10]. In this field, tendon-derived stem cells (TDSCs) appear to be very promising in equine regenerative medicine [11]. Actually, TDSCs have many advantages compared to BM-MSCs, such as more clonogenicity, a higher rate of proliferation, and multi-lineage differentiation potential. Moreover, the use of TDSCs decreases the risk for ectopic bone formation as reported with BM-MSC [12].

Usually, stem cells are isolated from biopsies performed on embryonic, foetal or adult living hosts. The restricted number of donors, and the limited amount of material obtained from biopsies are the two major limiting factors of this approach. In this context, stem cells harvested from cadaveric tissues represent a potential source for a multitude of even rare stem cells. This strategy should be explored and could generate new insights and provide future treatment strategies in regenerative medicine [13–17].

Taking these observations into account, fundamental and clinical research studies are now required to investigate whether MSCs isolated from equine cadavers are suitable, abundant, and readily available to be used in future clinical applications. The present work is the first, in both animal and human, to show that tissues harvested from cadaveric ligaments up to 72 hours post-mortem represent an available reservoir of specific stem cell populations.

## Methods

### Equine cadaver

Four cadaveric forelimbs, received within 48–72 hours post-mortem (from horses 18–20 years old) were used for this study. Horses were euthanised for reasons other than suspensory ligament (SL) injury and had no previous clinical history of forelimb lameness. SLs were confirmed free of any damage following examination of the tissues both macroscopically and microscopically. Consent for cadaver use was obtained from all horse owners, and all study procedures were approved by the Ethical Committee for Animal Use of the Faculty of Veterinary Medicine, University of Liege, Liege, Belgium.

### Cell isolation and culture

Tissue was harvested from the cores of SL branches. Three pieces (approximately 30–50 g) of tissue were recovered with a scalpel and forceps, cut into small pieces and processed immediately under aseptic conditions. Subsequently, the fragments were placed in plastic tubes (Corning Life Sciences, Corning, NY, USA) containing 0.2 % (w/v) collagenase A (Roche, Mannheim, Germany) in phosphate buffered saline (PBS, Lonza, Verviers, Belgium) supplemented with 1 % penicillin–streptomycin (Gibco*,* Invitrogen Corporation*,* Carlsbad, CA, USA)*,* and 1 % fungizone (Gibco)*.* They were incubated for 18 h at 37 °C in a humidified atmosphere containing 5.0 % CO_2_. After incubation, the remaining tissue pieces were removed and the digestion solution was filtered using a 40-μm-pore sized nylon cell strainer (BD Falcon*,* Franklin Lakes, NJ*,* USA). After digestion, the solution was centrifuged at 1000 rpm for 5 min and the supernatant discharged. The cell pellet was washed twice with PBS (Lonza), then resuspended and cultured in Dulbecco's modified Eagle's minimal essential medium (DMEM) with 4.5 g/L glucose (Lonza) supplemented with 10 % foetal bovine serum (FBS, Gibco), 1 % penicillin–streptomycin, 1 % fungizone and 1 % L-glutamine (Gibco) at 37 °C in a humidified atmosphere containing 5.0 % CO_2_. The medium was changed every three days until cell cultures reached confluence. Before passaging, cells were washed twice with PBS (Lonza), detached by using 0.05 % (w/v) trypsin in 0.1 % (w/v) ethylenediaminetetraacetic acid (EDTA, Lonza) and were either used in experiments or replated (1/3) in 25 cm^2^ culture flasks. At passage zero (P-0) cells were plated onto plastic culture dishes (Greiner Bio One, Frickenhausen, Germany) and from P1 to P20 in culture flasks (Nunclon*,* Roskilde*,* Denmark).

### Viability, population doubling, and freezing

Isolated EC-MSCs were cultured until subconfluent (70-80 %) and, at each passage, the percentage of cell viability was determined by mixing the cell suspension with 1:1 ratio of Trypan blue solution (Sigma Aldrich, St Louis, Mo., USA) for 2 min. Then, the cells were subsequently resuspended and viable cells (Trypan blue negative cells) were counted using a haemocytometer microchamber under a light microscope (Olympus IX71, Olympus*,* Tokyo*,* Japan).

The proliferative capacity of EC-MSCs was evaluated from P1 to P20 by Trypan blue exclusion assay. The cumulative population doubling (CPD) and population doubling time (PDT) were calculated using the following formulas:

$$ \begin{array}{c}\hfill \mathrm{C}\mathrm{P}\mathrm{D}=3.33\;\mathrm{X}\; log10\;\left(\mathrm{N}/\mathrm{N}0\right)\hfill \\ {}\hfill \mathrm{P}\mathrm{D}\mathrm{T}=\mathrm{C}\mathrm{T}/\mathrm{C}\mathrm{P}\mathrm{D}\hfill \end{array} $$

Where N0 is the initial cell seeding number, N is the cell harvest number and CT is the culture time.

For freezing, cells were suspended in freezing medium containing DMEM (Lonza) supplemented with 20 % FBS (Gibco) and 10 % dimethyl sulfoxide (DMSO, Sigma Aldrich). Samples were stored at −80 °C overnight and then placed in liquid nitrogen (−196 °C) for long*-*term storage. The thawing process involved placing the cryovial in a water bath at 37 °C to be thawed, and cryoprotectant was diluted immediately.

### Differentiation assay

Multipotent differentiation capability of EC-MSCs was assessed at P3. Cells intended to be used for osteogenic and adipogenic differentiation were seeded into 12-well plates at a density of 5 × 10^3^ cells/cm^2^ and 1 × 10^4^ cells/cm^2^, respectively. These cells were then incubated at 37 °C in a humidified atmosphere of 95 % air and 5 % CO2. After 24 h, the culture media were replaced by the differentiation media StemPro osteogenesis and StemPro adipogenesis (Gibco) and changed every three days. Non-induced EC-MSCs were cultured for the same time in growth medium as control. The osteogenic differentiation was assessed by Alizarin Red S staining for calcium deposits in extracellular matrix (Sigma-Aldrich). Adipogenic differentiation was checked by Oil Red O staining (Sigma-Aldrich) for the presence of lipid droplets in the cytoplasm.

For chondrogenic differentiation, a micromass culture method was achieved by seeding 5-μL droplets of 1.6 × 10^7^ viable cells/mL solution into the centre of 12-well plates. After 2 h under high humidity conditions, differentiation medium StemPro chondrogenesis (Gibco) was gently added and incubated at 37 °C with 5 % CO2. The differentiation medium was changed every three days. Non-induced EC-MSCs were cultured for the same time in growth medium as control. The chondrogenic differentiation was assessed by Alcian Blue staining (Sigma-Aldrich) for proteoglycan-rich extracellular matrix detection.

### Flow cytometric characterization

Flow cytometry was used to characterize the surface markers of the cultured EC-MSCs. Cells from P3 were dissociated from the growth flask using Hanks'-Based, Enzyme Free, Cell Dissociation Buffer (Gibco), and resuspended in DMEM (Lonza) containing 10 % FBS (Gibco). Samples were counted, centrifuged, and resuspended in PBS containing 10 % FBS. The cells were transferred to Eppendorf tubes at 5 × 10^5^ cells per 1 mL, washed twice with PBS containing 10 % FBS, and incubated for 1 h at room temperature in the dark with the following antibodies: PE-conjugated mouse anti*-*human CD73 (Clone AD2, BD Pharmingen)*,* PE-conjugated mouse anti-human CD90 (Clone 5E10, BD Pharmingen, Erembodegem, Belgium), PE-conjugated mouse anti-human CD105 (Clone 1G2, Beckman Coulter, Marseille, France), and FITC-conjugated rat anti-mouse CD45 (Clone 30-F11, eBioscience, Halle-Zoersel, Belgium). The samples were then washed twice with PBS, stained with 1 μL of Fixable Viability Dye eFluor® 450 per 1 mL of cells, vortexed, incubated for 30 min at 4 °C in the dark, and washed with PBS before analysing with flow cytometry. Unlabelled cells were used as the negative control for detection of autofluorescence.

### Cell cultures for immunofluorescence

Cells were plated on uncoated glass coverslips using a culture medium composed of DMEM (Lonza) with 1 % penicillin–streptomycin, 1 % fungizone, 1 % L-glutamine and 10 % FBS (Gibco) and then incubated for two to three days in an atmosphere of humidified 5 % CO_2_ in air.

### Fluorescent cell labelling

Before immunostaining, cells were fixed with 4 % (w/v) paraformaldehyde (Sigma Aldrich) in PBS (pH 7.4), at room temperature for 20 min, then permeabilised with 0.1 % Triton X-100 (Sigma Aldrich) in PBS at room temperature for 30 min. Non-specific binding was blocked for 1 h in 10 % goat serum (Sigma Aldrich) in PBS at room temperature. Cells were then incubated overnight at 4 °C with the following primary antibodies: rabbit anti-OCT-4 (1:200, Abcam, Cambridge, UK), mouse anti-SSEA- 1 (1:50, Santa Cruz Biotechnology, Santa Cruz, CA, USA), mouse anti-TUJ-1 (1:1500, Babco, Richmond, CA, USA), rabbit anti-TUJ-1 (1:1000, Covance, Cumberland, VA, USA), and rabbit anti-GFAP (1:1000, Dako, Glostrup, Denmark). After washing with PBS, the cells were incubated at room temperature for 1 h 30 min in the dark with appropriate fluorochrome-conjugated secondary antibodies: anti-rabbit Alexa Fluor 488 or 568 (1: 4000, Invitrogen, Grand Island, NY, USA), anti-mouse FITC (1:400, Santa Cruz Biotechnology), and anti-mouse Alexa Fluor 568 (1:4000, Molecular Probes, Leiden, Netherlands). Coverslips were washed with PBS, stained with 1:200 dilutions of 4',6-diamidino-2-phenylindole for 10 min (DAPI, Life Technologies, Carlsbad, CA, USA) for nuclear staining, and then mounted in fluorescence mounting medium and analysed under an Olympus IX71 microscope. Images were acquired using the microscope equipped with fluorescent optics, and (USB 2.0 CAMERA) software. The specificity of the antibodies was tested using an isotype control antibody and negative controls were obtained by omitting the primary antibody.

### Immunofluorescence staining quantification

To quantify the percentage of positively stained cells for OCT-4, SSEA-1, TUJ-1, and GFAP, the number of positive cells for each marker, in ten random fields of view, were counted and compared to the cell number positive for DAPI. An average value per marker was calculated. The percentages reported in this study refer to an average value (mean ± SD) of at least three experiments for each marker at P 3 and P20.

### Transmission electron microscopy (TEM) observation

Adherent cells were pre-fixed in the culture plate with 2.5 % (w/v) glutaraldehyde (Sigma Aldrich) solution in 0.1 M cacodylate buffer (Sigma Aldrich) pH 7.4 for 2 min at room temperature. Subsequently, cells were rinsed in the same buffer, scraped from the plate and centrifuged. The pellet was post-fixed for 30 min with a mixture of 1 % osmium tetroxide (OsO4) and 1.5 % potassium ferrocyanide (K4(Fe(CN)6)) in a 0.1 M cacodylate buffer (pH 7.4), dehydrated in a graded series of alcohols, and then embedded in EPON 812 (Fluka*,* Buchs*,* Switzerland) according to standard procedures. The resin specimen blocks were trimmed, ultrathin sections of selected areas were cut and mounted on copper grids, then contrasted with 1 % (w/v) uranyl acetate before examination with a Zeiss EM910 transmission electron microscope (Zeiss, Nanterre, France).

## Results

### Mesenchymal stem cells isolation, culture and morphological analysis

**Fig. 1:**
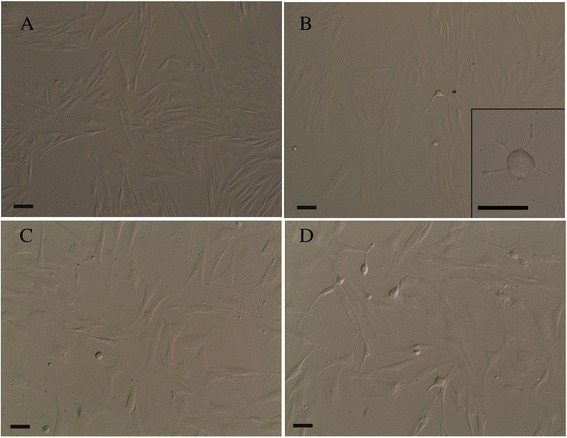
Phase contrast images of cultured EC-MSCs. Homogeneous fibroblast*-*like cells (spindle-shaped in appearance) at P0 (**a**); among fibroblast*-*like cells*,* some adherent cells with a different shape (spherical or oval) and size were distinguishable at P*2. Insert*: a spherical cell body with thin cytoplasmic extensions (**b**). Spherical cells without processes were free floating in the medium, whereas cells with neuronal- and glial-like morphology with extending processes were firmly adherent to the bottom of the flasks at P3 (**c**). Cells also showed unipolar, bipolar or multipolar extensions at P3 (**d**). Bars, 50 μm. *EC-MSCs* equine cadaver mesenchymal stem cells

Equine cadaver mesenchymal stem cells (EC-MSCs) were isolated from equine SL within 48–72 hours post-mortem. EC-MSCs were successfully expanded in vitro and maintained for 20 passages. Phase contrast microscopy of seeded cells revealed that cells adhered to plates after 48 h of the primary culture initiation. In our basic culture medium, cells at P0 were shown to be morphologically homogeneous fibroblast-like cells (Fig. 1a). From P1 to P20, cells with fibroblast-like appearance were the predominant population in the culture. Nevertheless, at P2, some adherent cells of different shape or size were distinguishable. Their cell bodies were spherical or oval with or without thin cytoplasmic extensions (Fig. 1b). Spherical cells without processes were free floating in the medium, whereas cells closely resembling neuronal and glial morphology with extended processes were firmly adherent to the bottom of the flasks (Fig. 1c). Other cells exhibiting unipolar, bipolar and multipolar extensions were also observed (Fig. 1d).

### Viability, population doubling and freezing

At each passage, cell viability analysis using Trypan blue assay revealed that the viability was usually greater than 96 %. During the period extending from P1 to P20, a high level of proliferation was recorded. The mean doubling time of the EC-MSCs in P1 was 23.5 ± 4.2 h and remained almost constant up to P20. After freezing in liquid nitrogen, the cells retained their ability to proliferate under the same culture conditions.

### Differentiation into osteocytes, adipocytes and chondrocytes

**Fig. 2:**
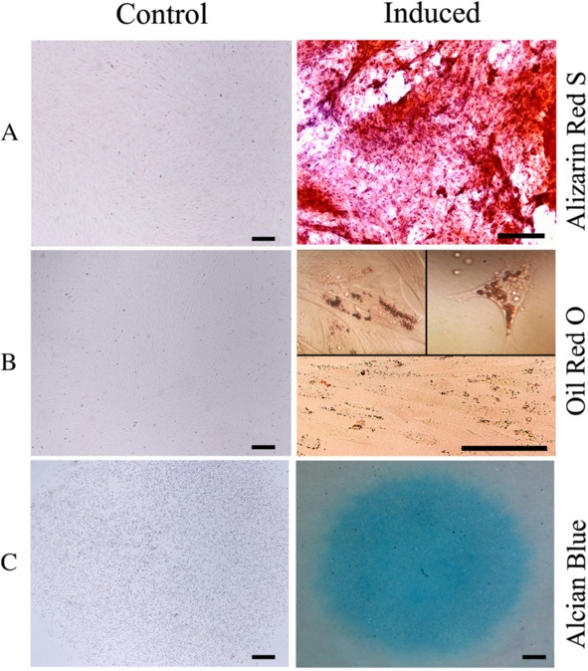
Tri-lineage differentiation potential of EC-MSC at P3. Extracellular calcium deposits stained with Alizarin Red S showed the differentiation of EC-MSC into the osteoblast lineage, whereas no calcium deposits were observed in non-induced cells (**a**). The accumulation of intracytoplasmic lipid droplets stained with Oil Red O indicated the adipogenic differentiation of EC-MSC, whereas no lipid droplets were observed in non-induced cells (**b**). Positive Alcian Blue staining of proteoglycan production confirmed the chondrogenic differentiation of EC-MSC, as compared to the absence of staining of control cells (**c**). Bars, 200 μm. *EC-MSC* equine cadaver mesenchymal stem cell

The multipotency of EC-MSCs was confirmed by tri-lineage differentiation assays. The differentiation of EC-MSCs towards osteogenic, adipogenic and chondrogenic lineages was shown after the 10^th^, 12^th^, and 15th day, respectively. Osteogenic differentiation was confirmed by positive Alizarin Red S staining of extracellular calcium deposits (Fig. 2a). Adipogenic differentiation was detected by the accumulation of intracellular lipid droplets into the cytoplasm using Oil Red O staining (Fig. 2b). Chondrogenic differentiation was confirmed by positive Alcian Blue staining, indicating proteoglycans deposition (Fig. 2c).

### Flow cytometric characterization

**Fig. 3:**
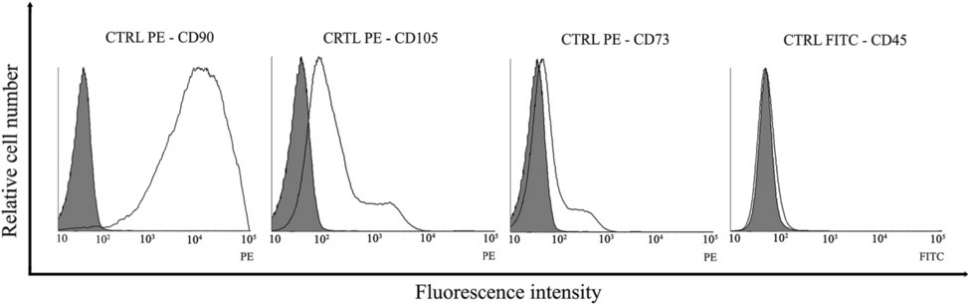
Flow cytometric analysis of cell-surface antigen expressions on cultured EC-MSCs. EC-MSCs were analysed at P3 for the cell surface markers CD90, CD73, CD105, and CD45. EC-MSCs showed high expression for CD90 (96.3 %), moderate expression for CD105 (64.31 %), and low expression for CD73 (23.09 %) while were negative for the leukocyte common antigen CD45. *EC-MSCs* equine cadaver mesenchymal stem cells

Fixable Viability Dye eFluor 450 distinguished between living and dead cells. A high percentage of viable cells (86.84 %) was seen and dead cells were excluded from the analysis. Flow cytometric analysis revealed that EC-MSCs were positive for CD90 (96.3 %) and CD105 (64.31 %), showed low expression of CD73 (23.09 %), and were negative for the leukocyte common antigen CD45 (Fig. 3).

#### Immunofluorescence staining and phenotypic characterization

##### Expression of pluripotency markers

**Fig. 4:**
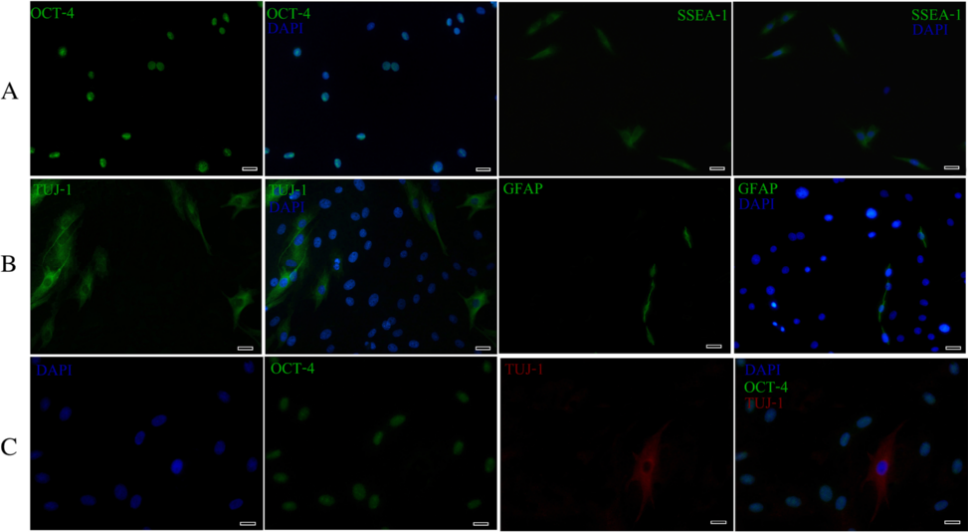
Immunocytochemistry analysis of pluripotency (OCT-4 and SSEA-1), neuronal (TUJ-1) and glial (GFAP) markers in cultured EC-MSCs at P3. High percentage of cells expressed OCT-4 and SSEA-1 (OCT-4, green; SSEA-1, green; DAPI, blue) (**a**). A fibrillar cytoplasmic staining was detected with TUJ-1 while the GFAP expression was seen in both the cytoplasm and the cytoplasmic extensions of only a few cells (TUJ-1, green; GFAP, green; DAPI, blue) (**b**). Analysis of TUJ-1 and OCT-4 localization showed that TUJ-1-positive cells exhibited a neuronal-like appearance and were OCT-4 negative (TUJ-1, red; OCT-4, green; DAPI, blue) (**c**). Bars: 20 μm. *OCT-4* pluripotent transcription factor, *SSEA-1* stage-specific embryonic antigen-1, *TUJ-1* neuron-specific class III beta-tubulin, *GFAP* glial fibrillary acidic protein, *EC-MSCs* equine cadaver mesenchymal stem cells, *DAPI* 4',6-diamidino-2-phenylindole

The stemness of cultured EC-MSCs was evaluated at P3 and P20 by using two markers, the pluripotent transcription factor (OCT-4) and the surface-based pluripotent marker (SSEA-1). OCT-4 immunofluorescence staining showed a clear nuclear localization. A high percentage of cells (Table 1) identified by DAPI staining expressed this marker. As illustrated, a high percentage of cells (Table 1) also demonstrated surface localization for SSEA-1(Fig. 4a; not shown for P20).

**Table 1:** Expression of pluripotent markers in EC-MSCs

Marker	Passage	
	P3	P20
OCT-4	86.6 ± 5.2	79.1 ± 8.2
SSEA-1	79.3 ± 0.5	77.3 ± 5.6

*OCT-4* pluripotent transcription factor, *SSEA-1* stage-specific embryonic antigen-1

The percentages refer to an average value (mean ± SD) of at least 3 experiments for each marker at P3 and P20

##### Expression of neuronal and glial markers

To assess the neuronal- and glial-like phenotypes of the cultured EC-MSCs, cells pre-labelled with DAPI staining, were analysed for expression of neuronal (TUJ-1) and glial (GFAP) specific markers at P1, P3 and P 20. Immunofluorescence microscopic analysis did not reveal any TUJ-1+ or GFAP+ cells at P1. On the contrary, approximately 15–16 % (Table 2) of the cell populations were TUJ-1-positive at P 3 and P 20 with a fibrillar cytoplasmic staining. In addition, GFAP expression has been shown in the cytoplasm and cytoplasmic extensions in a small number of cells (Table 2) at P3 and P20 (Fig. 4b; not shown for P20). Furthermore, TUJ-1 expression was evident in the cells negative for OCT-4 and displaying a neuronal-like morphology; generally they were pyramidal containing numerous thin neurite-like processes. The nucleus appeared to be round-to-oval in shape, centrally located where it occupied one half to one third of the cytoplasm area (Fig. 4c).

**Table 2:** Expression of neuronal and glial specific markers in EC-MSCs

Marker	Passage	
	P3	P20
TUJ-1	15 ± 0.8	16.1 ± 7.9
GFAP	4.3 ± 0.5	5.8 ± 0.9

EC-MSCs equine cadaver mesenchymal stem cells, *TUJ-1* neuron-specific class III beta-tubulin, *GFAP* glial fibrillary acidic protein

The percentages refer to an average value (mean ± SD) of at least 3 experiments for each marker at P3 and P20

### Ultrastructural characterization (TEM)

**Fig. 5:**
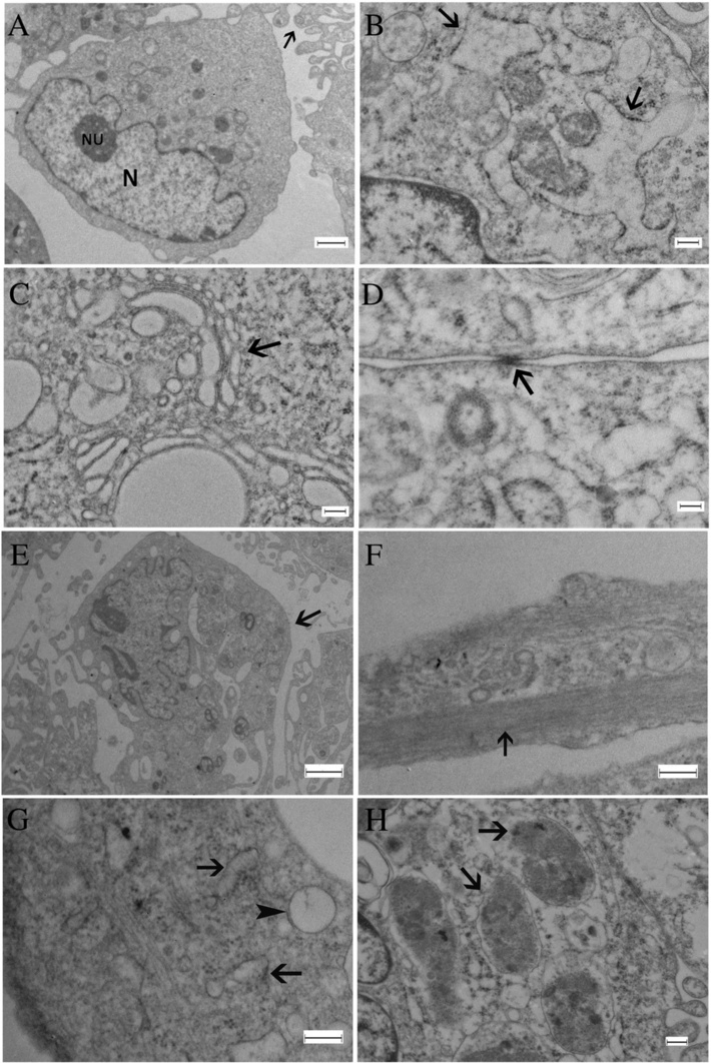
Electron micrographs illustrating ultrastructural characteristics of EC-MSCs at P1 and neuron-like cells derived from cultured EC-MSCs at P3. EC-MSCs at P1 have large nuclei (N), prominent nucleoli (NU), small pseudopodia-like processes at the cell periphery (*arrow*) (**a**). Dilated rough endoplasmic reticulum (*arrows*) (**b**) and Golgi complexes (*arrow*) (**c**) were present in cytoplasm. Tight junctions were seen in the periphery (*arrow*) (**d**). Neuron-like cells at P3 have outgrowing extensions (*arrow*) (**e**). These extensions contain bundles of microtubules, which run parallel to their long axis (*arrow*) (**f**). Endoplasmic reticulum (*arrow*) and vesicles (*arrowhead*) (**g**). Residual bodies (lipofuscin pigment granules) were present within the cytoplasm (*arrow*) (**h**). Bar in (**a**): 2 μm; bars in (**b**, **c**, **d**, **f**, **h**): 200 nm; bar in (**e**): 5 μm; bar in (**g**): 100 nm. *EC-MSCs* equine cadaver mesenchymal stem cells

Ultrastructural analysis revealed that the EC-MSCs are homogeneous cell populations at P1. Cells had large nuclei with a prominent nucleolus, and an abundant amount of euchromatin. Small pseudopodia-like processes around the cell periphery were also observed (Fig. 5a). Organelles, such as dilated rough endoplasmic reticulum (Fig. 5b) and Golgi complexes (Fig. 5c) were present in cytoplasm. In addition, tight junctions were observed between these cells (Fig. 5d). However, some cells at P3 and P20 appeared to have outgrowing extensions (Fig. 5e) containing bundles of microtubules, aligned parallel to their long axis (Fig. 5f), endoplasmic reticulum, neurofilaments and vesicles (Fig. 5g). Moreover, residual bodies (lipofuscin pigment granules) were also visible within the cytoplasm of these cells (Fig. 5h).

## Discussion

Mesenchymal stem cells (MSCs) have immense potential in tissue engineering and are commonly used in equine clinical medicine [2, 3]. The possibility that cadavers may represent, for tissue maintenance and regeneration, an alternative source of stem cells, is very exciting. Traditionally, it was thought that 48 hours after death, the presence of these stem cells in a necrotic microenvironment led them to lose their utility and potential benefits for experimental and clinical applications [13]. Actually, while the differentiated cells die within two days after death, the MSCs reside in their hypoxic tissue niche in a state of quiescence or dormancy, and survive by adapting to a low rate of oxygen consumption with a slow metabolism and deactivated transcription [18, 19]. Consistently, anoxia and a lack of nutrients participate in the positive selection of more robust and undifferentiated stem cells [17]. The use of cadaveric materials would prevent the need for foetal, embryonic or healthy living donor sources that may help to avoid serious ethical considerations in regenerative medicine applications [13].

To the best of our knowledge, this work is the first, in both animal and human, to show that it is indeed possible to isolate viable MSCs from cadaveric ligament up to 72 hours post-mortem. In the current study, samples were taken from the SL branches which are composed only of dense connective tissue [20]. EC-MSCs were successfully isolated, expanded and then maintained for 20 passages with high cell viability and proliferation. In agreement with the International Society for Cellular Therapy criteria for defining MSCs, the data presented here reveal that cultured EC-MSCs display characteristic MSC cell surface markers. They showed high expression for CD90 (96.3 %), moderate expression for CD105 (64.31 %), and low expression for CD73 (23.09 %) while they were negative for the leukocyte common antigen CD45. These results are consistent with several previous studies that have demonstrated a variable expression of CD105 and CD73 for equine MSC [21–27]. Interestingly, EC-MSCs were able to prove their tri-lineage potential and differentiated towards osteoblast, adipocyte and chondrocyte. Moreover, the stemness of the cultured EC-MSCs was assessed by analysing their expression profile for octamer-binding transcription factor 4 (OCT-4) and stage-specific embryonic antigen 1 (SSEA-1). In fact, OCT-4, a master nuclear transcriptional regulator, and SSEA-1, a cell surface antigen, are currently associated with undifferentiated stem cells and together play an essential role in maintaining pluripotency and stem cell renewal [28–31]. Previous reports in equine species demonstrated that adult MSCs derived from umbilical cords (UCB) express OCT-4 and SSEA-1, while MSCs derived from AD and BM express only OCT-4 [32, 33]. In this study, a high number of EC-MSCs expressed OCT-4 within the nucleus, and SSEA-1 on the cell surface. These findings confirm that a high percentage of EC-MSCs are naive and undifferentiated sharing ancestral or primitive characteristics [17, 29].

Certain cytokines, growth factors and chemical inducers are commonly used to differentiate MSCs into multiple mesenchymal lineages, including osteoblasts, chondrocytes, adipocytes, myocytes and tenocytes, as well as hepatocytes in endoderm and neural cells in ectoderm [34–36]. Surprisingly, in our cell culture conditions, in the absence of any kind of differentiation stimuli, some cells derived from EC-MSCs displayed a typical neuronal and glial-like morphology. As their expressions are regarded to be evidence of MSC predisposition to differentiate into a neuronal lineage, neuronal (TUJ-1) and glial (GFAP) specific markers were evaluated in our cultured EC-MSCs [37]. TUJ-1, a neuron-specific class III beta-tubulin, is a major component of neuronal microtubules. Its expression has been considered as one of the earliest markers of neuronal commitment in primitive neuroepithelium, as well as essential for axon growth and guidance during normal brain development [38, 39]. GFAP, a class III IF protein, is found in glial cells such as astrocytes and Schwann cells. Furthermore, its expression is seen as the hallmark of astrocytic differentiation [40]. Interestingly, immunofluorescence analysis confirmed our observations under phase contrast microscopy, showing that approximately 15 % of the cell populations were TUJ-1-positive with a fibrillar cytoplasmic staining among the cells displaying neuronal-like morphology, whereas the GFAP expression was detected in both the cytoplasm and the cytoplasmic extensions of only a few cells. These results obtained from immunofluorescence analysis are in line with our TEM observations, revealing that EC-MSCs from P1 have MSC characteristics as previously described [41–43]. Nonetheless, some of these cells exhibited ultrastructural features characteristic of neuronal cell bodies and processes at P3 and P20. These observations are also in accordance with another study on ultrastructural changes of MSCs during neuronal induction [44].

## Conclusions

This study demonstrated that a viable stem cell population can be isolated and expanded from equine cadaver ligaments up to 72 hours post-mortem. Differentiation assays, flow cytometry, immunofluorescence and TEM analyses confirmed the stemness of these cells. Our research may benefit the development of an advantageous source of stem cells for regenerative medicine and cell therapy technologies. EC-MSCs may provide a low cost solution in ready supply for the development of novel therapeutic strategies in horses. Although the generation of MSCs into neuronal lineage with specific inductions is well-documented, our findings herein indicate that some EC-MSCs, without any specific external stimulus, are able to spontaneously express in vitro neural and glial markers. However, the functional recovery of these cells should be verified. In a more specific way, inducing differentiation of EC-MSCs into tenocytes would appear to be a promising approach for the treatment of equine tendinopathies.

## Abbreviations

AD-MSCs: Adipose-derived mesenchymal stem cells
BM-MSCs: Bone marrow-derived mesenchymal stem cells
CT: Culture time
CPD: Cumulative population doubling
EC-MSCs: Equine cadaver mesenchymal stem cells
DMSO: Dimethyl sulfoxide
DAPI: 4',6-diamidino-2-phenylindole
EDTA: Ethylenediaminetetraacetic acid
DMEM: Dulbecco's modified Eagle's minimal essential medium
FBS: Foetal bovine serum
FITC: Fluorescein isothiocyanate
GFAP: Glial fibrillary acidic protein
N0: Initial cell seeding number
N: Cell harvest number
OCT-4: Pluripotent transcription factor
PDT: Population doubling time
P: Passage
PBS: Phosphate-buffered saline
PE: Phycoerythrin
SSEA-1: Stage-specific embryonic antigen-1
TUJ-1: Neuron-specific class III beta-tubulin
TEM: Transmission electron microscope
TDSCs: Tendon-derived stem cells
SL: Suspensory ligament
